# Majocchi's granuloma of the scalp caused by *Trichophyton tonsurans* in an immunosuppressed adolescent with Crohn's disease: a case report

**DOI:** 10.3389/fped.2026.1921632

**Published:** 2026-09-03

**Authors:** Gaja Turk, Jakub Hurych, Daniela Lzicarova, Eliska Onderkova, Miroslav Koblizek, Lydia Paroulkova, Tereza Lerchova, Ivana Copova, Ondrej Hradsky, Pavel Drevinek

**Affiliations:** 1 Department of Medical Microbiology, Second Faculty of Medicine, Charles University and Motol and Homolka University Hospital, Prague, Czechia; 2 Department of Pathology and Molecular Medicine, Second Faculty of Medicine, Charles University and Motol and Homolka University Hospital, Prague, Czechia; 3 Department of Dermatology, Motol and Homolka University Hospital, Prague, Czechia; 4 Department of Paediatrics, Second Faculty of Medicine, Charles University and Motol and Homolka University Hospital, Prague, Czechia

**Keywords:** case report, Crohn's disease, dermatophytes, immunosuppression, Majocchi's granuloma, *Trichophyton tonsurans*

## Abstract

**Introduction:**

Majocchi's granuloma (MG) is a rare invasive dermatophytosis characterised by invasion of hair follicles and perifollicular granulomatous inflammation. Scalp involvement is uncommon, and *Trichophyton tonsurans* is an unusual aetiological agent of MG.

**Patient concerns and clinical findings:**

A 16-year-old boy with Crohn's disease treated with infliximab and azathioprine developed a painful inflammatory occipital scalp lesion with colliquation, pustulation, and regional lymphadenopathy shortly after a haircut-related injury.

**Diagnostic assessment:**

Initial antibacterial and antiviral therapy was ineffective. Dermatological assessment suggested an unusually severe tinea capitis/kerion-like presentation. Biopsy showed periodic acid–Schiff-positive fungal elements in the dermis with granulomatous suppurative inflammation. Culture yielded a weakly growing dermatophyte consistent with *Trichophyton* sp. Bidirectional sequencing of the ITS2 region showed 100% similarity to members of the *Trichophyton mentagrophytes* series and did not discriminate between *T. tonsurans* and *Trichophyton equinum*; *T. tonsurans* was considered the most likely species based on the combined clinical and epidemiological context.

**Therapeutic intervention and outcomes:**

Terbinafine 250 mg once daily failed to control disease progression. The patient improved after switching to itraconazole 200 mg twice daily, combined with repeated surgical debridement, topical antifungal treatment, and temporary interruption of immunosuppression. The lesion healed completely and hair regrowth was restored.

**Conclusion:**

This case highlights the need for early microbiological and histopathological assessment of severe scalp lesions in immunosuppressed patients, especially after skin-barrier disruption. It also raises awareness of possible dermatophyte transmission related to hairdressing and of a lack of clinical response to terbinafine in dermatophyte infections.

## Introduction

Dermatophytes primarily infect superficial keratinised tissues; however, they may rarely penetrate deeper layers and cause invasive disease (1). Majocchi's granuloma (MG) is an uncommon invasive form of dermatophytosis defined by dermatophyte hyphae within hair follicles and the dermis, with perifollicular or dermal granulomatous inflammation (2). Predisposing factors include pre-existing superficial dermatophytosis, disruption of the skin barrier, and local or systemic immunosuppression (1).

Herein, we report a rare case of scalp MG in an immunosuppressed adolescent with Crohn's disease (CD) after haircut-related trauma. The case is notable because scalp MG is uncommon, *T. tonsurans* is a rare cause of MG, and the clinical course was complicated by a repeated lack of response to terbinafine therapy.

## Case presentation

### Patient information

A 16-year-old boy with CD was receiving combination immunosuppression with infliximab and azathioprine. He also had a history of atopic dermatitis treated locally with corticosteroids. No pre-existing superficial dermatophytosis was reported before the episode. In early July 2020, he sustained a minor injury to the occipital scalp during a haircut. Shortly thereafter, he had close contact with his grandmother's cat. Several family members developed dermatophytosis around the same period, although these infections were not microbiologically confirmed.

### Clinical findings

Within approximately 10–14 days of the haircut-related injury, the patient developed a painful inflammatory occipital scalp lesion with colliquation and regional cervical lymphadenopathy. The lesion progressed despite empirical therapy and developed draining pustules extending towards the right parieto-occipital region. A dermatological assessment suggested tinea capitis with an unusually severe kerion-like presentation in the context of immunosuppression.

### Diagnostic assessment

**Figure 1:**
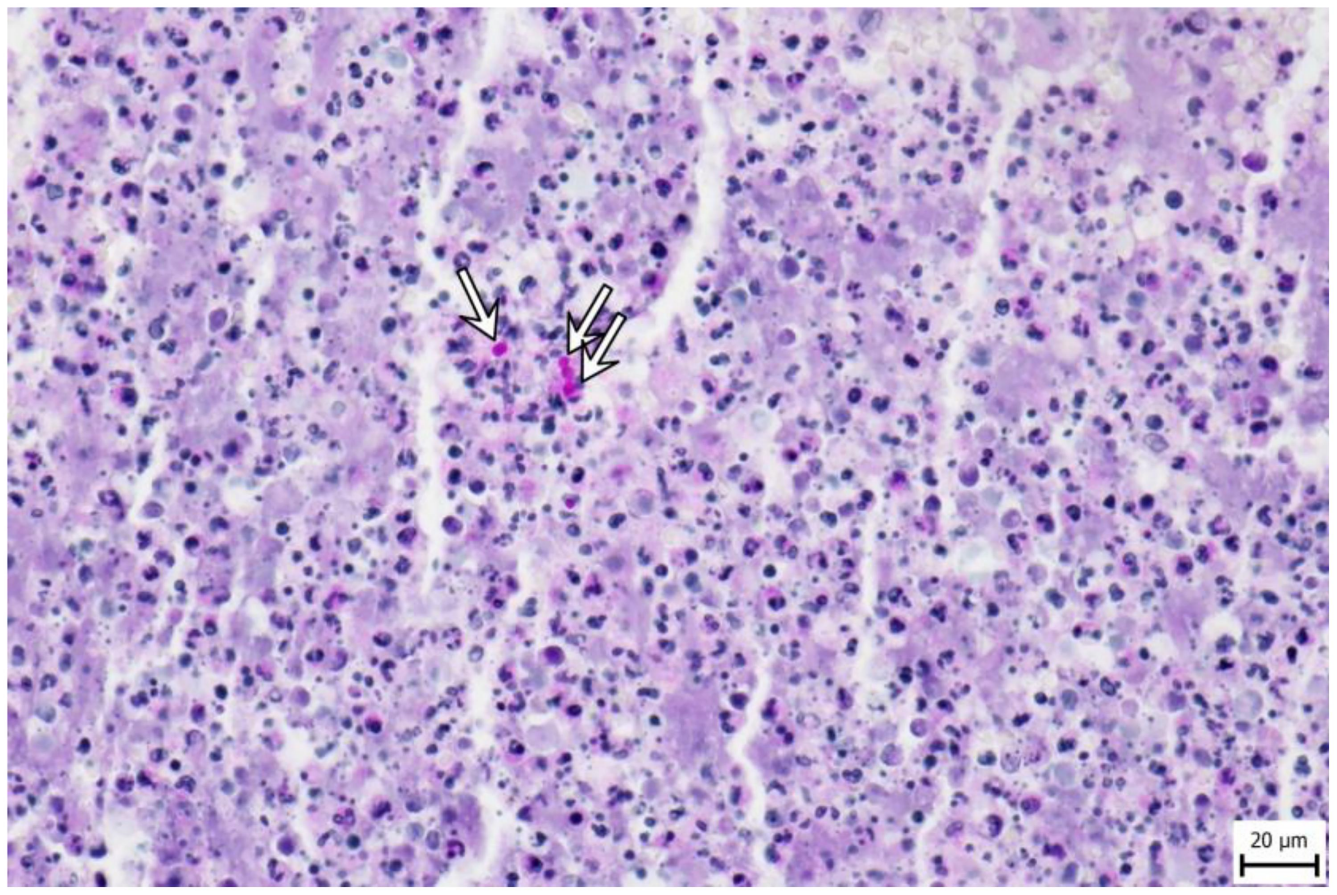
Histopathological evidence of invasive dermatophytosis. Periodic acid–Schiff staining demonstrates magenta fungal elements within the dermis (arrows) in a background of granulomatous suppurative inflammation. Scale bar, 20 μm.

**Figure 2:**
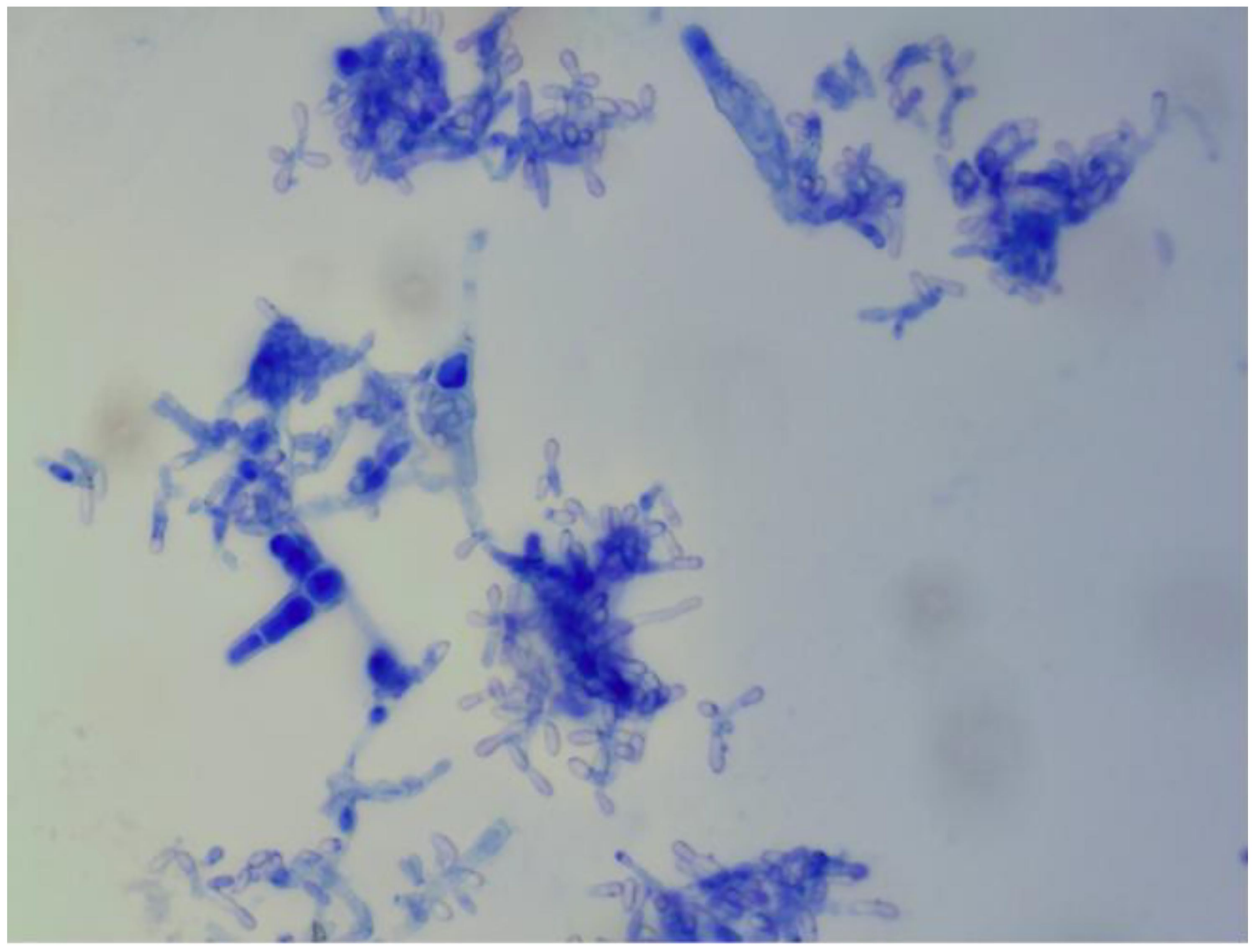
Microscopic morphology of the weakly growing dermatophyte culture. An impression preparation stained with lactophenol cotton blue shows septate fungal hyphae compatible with *Trichophyton* sp. The isolate subsequently failed to grow on subculture, precluding additional phenotypic or molecular testing.

Aspirate, hair, skin, and biopsy samples were obtained for mycological and histopathological assessment. Histopathology demonstrated periodic acid–Schiff (PAS)-positive fungal elements in the dermis associated with granulomatous suppurative inflammation, supporting the diagnosis of MG (Figure 1). Culture yielded a weakly growing filamentous fungus with morphological features consistent with a dermatophyte and *Trichophyton* (Figure 2); however, the isolate could not be maintained on subculture. Direct 18S rDNA sequencing confirmed the family Arthrodermataceae. Bidirectional sequencing of the ITS2 region was performed, and the forward and reverse sequences were analysed using NCBI BLAST and MycoBank, which showed 100% similarity to the *Trichophyton mentagrophytes* series, with equivalent matching to *T. tonsurans* and *T. equinum*. The ITS2 marker therefore did not permit definitive species-level discrimination. On the basis of the predominantly anthropophilic ecology of *T. tonsurans*, the haircut-related exposure, reports of contemporaneous dermatophytosis in family members, and the absence of a confirmed equine source, *T. tonsurans* was considered the most likely species; nevertheless, the aetiological assignment remains probabilistic rather than definitive.

The main diagnostic challenge was the unusual scalp location and the initially bacterial- or viral-appearing inflammatory presentation. Differential diagnoses included bacterial scalp infection, severe tinea capitis/kerion celsi, herpesvirus infection, and invasive dermatophytosis. The diagnosis of MG was based on the demonstration of fungal structures invading the dermis in association with granulomatous suppurative inflammation, together with compatible culture and molecular findings. Additional multilocus sequencing using translation elongation factor 1 or beta tubulin (BT2), antifungal susceptibility testing, and SQLE (squalene epoxidase gene) sequencing could not be performed because the original isolate failed to grow on subculture and no viable culture was retained.

### Therapeutic intervention

Initial empirical treatment with cefuroxime and subsequently oxacillin was ineffective. A short course of intravenous aciclovir was stopped after herpes simplex virus PCR from the lesion was negative. Oral terbinafine 250 mg once daily was used during the diagnostic phase and was continued/reintroduced after dermatophytosis was suspected; however, the disease continued to progress. Treatment was therefore switched to itraconazole 200 mg twice daily. Systemic antifungal therapy was complemented by repeated surgical drainage/debridement and topical antifungal treatment. Azathioprine, and subsequently other immunosuppressive treatments as clinically appropriate, were temporarily interrupted because of the severity and progression of infection. The clinical course is summarised in Table 1.

**Table 1:** Clinical timeline.

Date/interval	Clinical course	Diagnostic phase	Therapeutic phase
1 July 2020	Minor occipital scalp injury during haircut	—	—
3 July 2020	Close contact with grandmother's cat; family dermatophytosis later reported	—	—
11–14 July 2020	Painful occipital lesion with colliquation and cervical lymphadenopathy	—	Oral cefuroxime started
From 18 July 2020	Hospitalised because of progression; CRP ∼50–77 mg/L	Swab culture and serology—non-diagnostic	IV cefuroxime; later oxacillin for suspected superinfection
23–27 July 2020	Draining pustules with parieto-occipital extension	HSV PCR negative	Terbinafine started; brief IV aciclovir, then stopped; oxacillin ineffective
27 July 2020	Dermatology suspected severe tinea capitis/kerion	Biopsy, aspirate, hair, and skin samples collected	Azathioprine withheld; drainage/debridement performed
30 July 2020	Progression on terbinafine, then improvement after treatment change	PAS-positive dermal fungi with granulomatous suppuration; *Trichophyton*-like dermatophyte in microscopy	Switched to itraconazole 200 mg twice daily; topical therapy and repeated debridement
3 August 2020	Discharged from hospital	ITS2 sequencing: 100% match to *T. mentagrophytes* series; *T. tonsurans*/*T. equinum* unresolved	Itraconazole daily for 6 weeks, then alternate-week therapy for 4 months
26 November 2020	Follow-up: Complete healing and restored hair growth	—	Antifungal therapy stopped

Diagnostic and therapeutic events are presented in separate columns to clarify their temporal relationship. CRP, C-reactive protein; HSV, herpes simplex virus; ITS2, internal transcribed spacer 2, PAS, periodic acid–Schiff.

### Follow-up and outcomes

**Figure 3:**
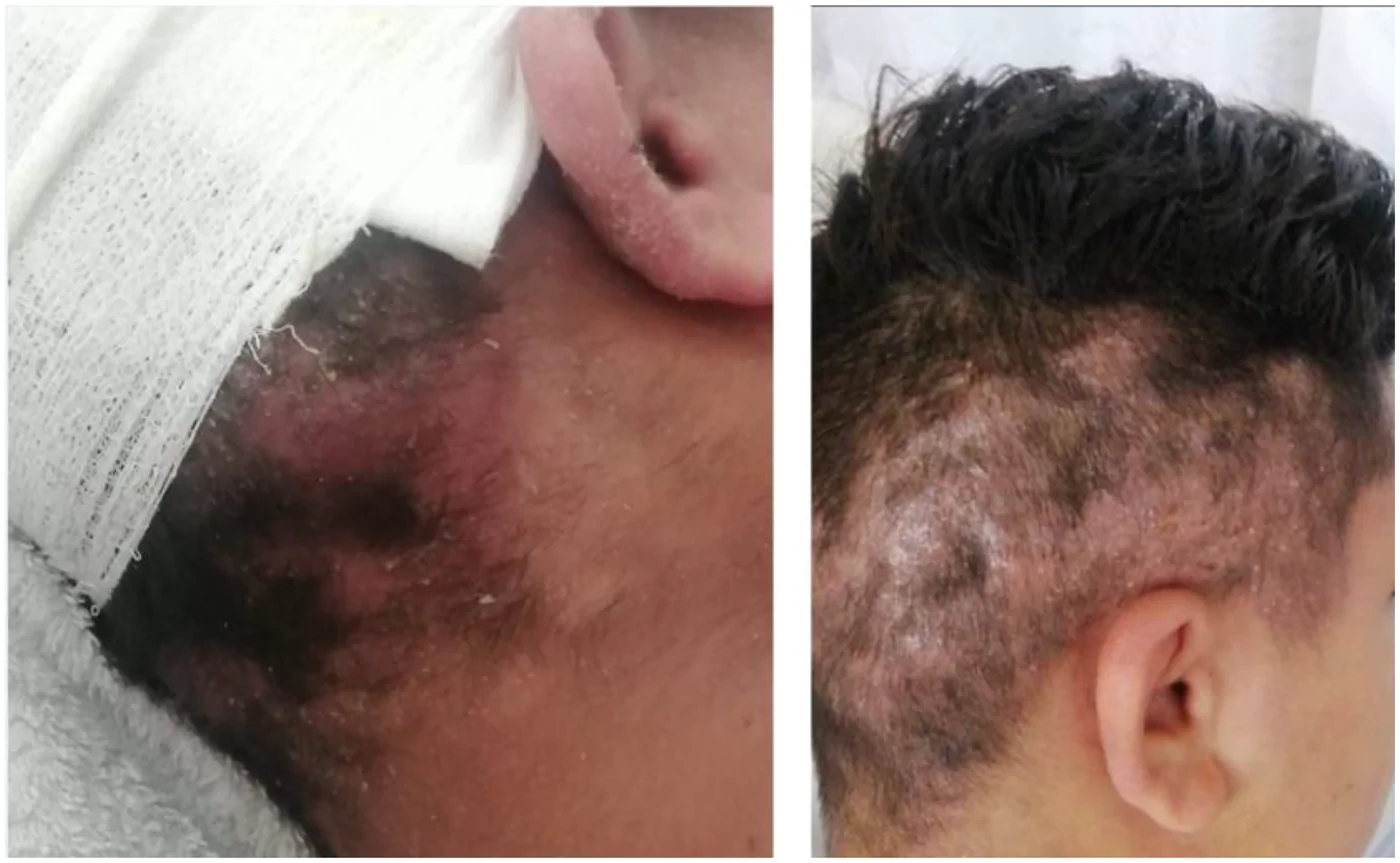
Clinical manifestation of the occipital scalp lesion.

Clinical improvement became apparent after the initiation of itraconazole as part of a multimodal treatment strategy. After 6 weeks of daily treatment, itraconazole was continued in an alternate-week regimen for an additional 4 months. The lesion healed completely, and normal hair growth resumed (Figure 3). No permanent alopecia was reported. The clinical course was compatible with terbinafine treatment failure despite reported adherence and adequate dosing, but microbiological resistance was not proven. No major adverse events related to itraconazole were documented.

### Patient perspective

The patient and his family appreciated the eventual identification of the cause after several ineffective empirical treatments and were particularly relieved by complete healing and restoration of hair growth. Written consent for publication of the clinical details and images was obtained from the patient's legal guardian.

## Discussion

MG most commonly affects the lower extremities and only rarely involves the scalp (1). In the present case, haircut-related trauma was a plausible trigger because disruption of the skin barrier may allow dermatophytes to enter deeper skin layers. The patient's treatment with azathioprine and infliximab, a tumour necrosis factor *α* inhibitor, likely further contributed to the unusually severe clinical course, as effective control of dermatophyte infection depends substantially on cellular immune responses (3, 4). Local corticosteroid exposure for atopic dermatitis may have provided an additional local predisposing factor.

Kerion celsi and MG may overlap clinically, especially on the scalp. Kerion is a highly inflammatory form of tinea capitis centred on infected hair follicles, whereas MG requires histological evidence of dermatophyte invasion beyond the superficial epidermis into the follicular/perifollicular dermis, accompanied by granulomatous inflammation. In this case, PAS-positive fungal elements within the dermis, together with granulomatous suppurative inflammation, supported classification as MG rather than uncomplicated kerion.

The usual causative agent of MG is *Trichophyton rubrum* (1), whereas *T. tonsurans*, although well recognised in tinea capitis and tinea gladiatorum, is a rare cause of MG (2, 5). We found no reported cases of MG caused by *T. equinum*. However, ITS2 sequencing in the present case could not distinguish *T. tonsurans* from *T. equinum*, and the title and conclusions therefore reflect that *T. tonsurans* was the most likely, rather than definitively proven, species. The cat exposure was considered a less likely source because *T. tonsurans* is predominantly anthropophilic, although rare animal infections have been described (6, 7). Hairdressing tools were considered a plausible source because outbreaks and clusters of *T. tonsurans* infections linked to barbershops or hair salons have been reported, and contamination of hairdressing tools with anthropophilic dermatophytes has been documented (5, 8, 9). Nevertheless, neither the family members, the cat, nor the hairdressing equipment were microbiologically sampled, and the transmission route remains unconfirmed.

The reason for the lack of clinical response to terbinafine remains uncertain. Terbinafine is generally used as first-line therapy for dermatophyte infections in Czechia (10), but terbinafine-resistant dermatophytes have increased globally, particularly with the spread of *T. indotineae* (11). Resistance is commonly associated with substitutions in the SQLE gene. Reduced susceptibility and SQLE mutations have also been reported in *T. tonsurans* (12, 13). In the present case, however, neither antifungal susceptibility testing nor SQLE sequencing was possible because the isolate failed on subculture and no viable culture remained. Consequently, the observed clinical failure cannot be equated with proven microbiological resistance.

Alternative explanations include the high fungal burden, deep granulomatous and suppurative disease requiring source control, ongoing immunosuppression, variable oral exposure, and possible species-related differences. Altered pharmacokinetics in CD could theoretically affect oral antifungal exposure (14), although subsequent response to oral itraconazole makes major global malabsorption less likely. Importantly, improvement occurred after a combination of itraconazole, repeated debridement, topical treatment, and temporary reduction of immunosuppression; therefore, the therapeutic success cannot be attributed to itraconazole alone.

This study has several strengths. The diagnosis was supported by histopathology, culture, direct molecular testing, and multidisciplinary assessment, and the newly added histopathological and microbiological figures provide direct visual documentation. Limitations include the inability to obtain definitive multilocus species identification, lack of antifungal susceptibility testing and SQLE sequencing, absence of microbiological confirmation in family members, and absence of formal testing of the suspected environmental source.

The main take-away message is that severe scalp lesions in immunosuppressed patients, particularly after skin-barrier disruption, should prompt early dermatological, microbiological, and histopathological evaluation. Early recognition of invasive dermatophytosis can prevent prolonged ineffective antibacterial therapy, guide appropriate antifungal treatment and source control, and reduce the risk of complications such as permanent hair loss or disseminated infection.

## Data Availability

The raw data supporting the conclusions of this article will be made available by the authors, without undue reservation.
