# Supplementary material for: Majocchi's granuloma of the scalp caused by Trichophyton tonsurans in an immunosuppressed adolescent with Crohn's disease: a case report
Source: Front Pediatr. 2026 Sep 3;14:1921632. doi: 10.3389/fped.2026.1921632 (PMC13582395; doi:10.3389/fped.2026.1921632)
Supplement: Supplementary file 1 [file Supplementaryfile1.docx]

**INFORMOVANÝ SOUHLAS ZÁKONNÉHO ZÁSTUPCE S PUBLIKACÍ KAZUISTIKY**

**a klinické obrazové dokumentace v anonymizované podobě**

Název kazuistiky / pracovního sdělení: Majocchiho granulom vlasaté části hlavy způsobený *Trichophyton tonsurans* u imunokompromitovaného adolescenta s Crohnovou chorobou

Pracoviště: Fakultní nemocnice Motol a Homolka

Kontaktní osoba: Jakub Hurych, M.D., Ph.D.; e-mail: jakub.hurych@lfmotol.cuni.cz

# Údaje o pacientovi

Jméno a příjmení pacienta: _______________________________________________

Datum narození pacienta: ______________________

Jméno a příjmení zákonného zástupce: ___________________________________

Vztah k pacientovi: ______________________

# Informace o publikaci

Byl/a jsem srozumitelně informován/a, že ošetřující a odborný tým připravuje odbornou kazuistiku, tedy popis jednoho klinického případu, určenou k publikaci v odborném časopise a/nebo k odborné prezentaci. Účelem publikace je vzdělávání zdravotnických pracovníků a sdílení medicínských poznatků.

Kazuistika může obsahovat anonymizované údaje o zdravotním stavu pacienta, průběhu onemocnění, provedených vyšetřeních, výsledcích laboratorních a histologických vyšetření, použité léčbě a jejím výsledku. Součástí publikace mohou být také klinické fotografie postižené oblasti, budou-li upraveny a vybrány tak, aby pacienta nebylo možné přímo identifikovat.

V publikaci nebude uvedeno jméno ani příjmení pacienta, rodné číslo, přesné datum narození, adresa ani jiné přímé identifikační údaje. Beru však na vědomí, že úplnou anonymitu nelze ve všech případech absolutně zaručit, zejména pokud by někdo znal podrobnosti zdravotního stavu pacienta z jiného zdroje.

Publikace může být zveřejněna v tištěné i elektronické podobě, včetně otevřeně dostupných internetových databází odborné literatury. Po zveřejnění již nemusí být možné publikaci zcela stáhnout ze všech zdrojů, databází nebo kopií.

# Dobrovolnost souhlasu

Souhlas s publikací je dobrovolný. Odmítnutí souhlasu nemá žádný vliv na současnou ani budoucí zdravotní péči o pacienta. Souhlas mohu odvolat kdykoliv před odesláním rukopisu k publikaci nebo před jeho zveřejněním. Po zveřejnění publikace již nemusí být možné odvolání souhlasu plně technicky provést.

Za poskytnutí souhlasu nevzniká pacientovi ani zákonnému zástupci nárok na finanční odměnu. Publikace bude použita výhradně k odborným, vědeckým a vzdělávacím účelům.

# Prohlášení zákonného zástupce

□ Potvrzuji, že jsem měl/a možnost položit doplňující otázky a že mi byly zodpovězeny srozumitelně.

□ Rozumím tomu, jaké informace a případně obrazová dokumentace mohou být publikovány.

□ Souhlasím s publikací anonymizované kazuistiky pacienta v odborném časopise a/nebo v rámci odborné prezentace.

□ Souhlasím s publikací anonymizované klinické obrazové dokumentace, pokud bude použita tak, aby pacienta nebylo možné přímo identifikovat.

Poznámka / omezení souhlasu (nepovinné):

___________________________________________________________________________

___________________________________________________________________________

___________________________________________________________________________

# Podpisy

Místo: ________________________________	Datum: ________________________

Podpis zákonného zástupce: ______________________________________________

Jméno a podpis zdravotnického pracovníka, který souhlas převzal:

___________________________________________________________________________

Je-li to s ohledem na věk a rozumovou vyspělost pacienta vhodné, doporučuje se zaznamenat také souhlas/nesouhlas pacienta:

Vyjádření pacienta: □ souhlasím □ nesouhlasím

Podpis pacienta: ________________________________	Datum: ______________
